# Genome-Wide Association Study of Phytic Acid in Wheat Grain Unravels Markers for Improving Biofortification

**DOI:** 10.3389/fpls.2022.830147

**Published:** 2022-02-15

**Authors:** Zhengyu Wen, Philomin Juliana, Harshaant S. Dhugga, Mario Pacheco, Ulises I. Martínez, Agustin Aguilar, Maria I. Ibba, Velu Govindan, Ravi P. Singh, Kanwarpal S. Dhugga

**Affiliations:** International Maize and Wheat Improvement Center (CIMMYT), El Batan, Mexico

**Keywords:** biofortification, grain nutrition, iron, zinc, phytic acid, GWAS

## Abstract

Biofortification of cereal grains offers a lasting solution to combat micronutrient deficiency in developing countries where it poses developmental risks to children. Breeding efforts thus far have been directed toward increasing the grain concentrations of iron (Fe) and zinc (Zn) ions. Phytic acid (PA) chelates these metal ions, reducing their bioavailability in the digestive tract. We present a high-throughput assay for quantification of PA and its application in screening a breeding population. After extraction in 96-well megatiter plates, PA content was determined from the phosphate released after treatment with a commercially available phytase enzyme. In a set of 330 breeding lines of wheat grown in the field over 3 years as part of a HarvestPlus breeding program for high grain Fe and Zn, our assay unraveled variation for PA that ranged from 0.90 to 1.72% with a mean of 1.24%. PA content was not associated with grain yield. High yielding lines were further screened for low molar PA/Fe and PA/Zn ratios for increased metal ion bioavailability, demonstrating the utility of our assay. Genome-wide association study revealed 21 genetic associations, six of which were consistent across years. Five of these associations mapped to chromosomes 1A, 2A, 2D, 5A, and 7D. Additivity over four of these haplotypes accounted for an ∼10% reduction in PA. Our study demonstrates it is possible to scale up assays to directly select for low grain PA in forward breeding programs.

## Introduction

Biofortification of cereal grains is an effective route to mitigate malnourishment of underprivileged populations in developing countries (Nestel et al., 2006; Bouis and Saltzman, 2017). Most breeding efforts toward this goal have been focused on increasing the concentrations of micronutrients like iron (Fe) and zinc (Zn) in the grain (Velu et al., 2016, 2018; Cu et al., 2020). Yet, the bioavailability of these ions is known to be reduced by phytic acid (PA), which also accumulates in the grain (Turnlund et al., 1984; Hallberg et al., 1989; Roohani et al., 2013). Once chelated by PA, these metal micronutrient ions pass through the digestive tract without being absorbed (Roohani et al., 2013).

Antinutritional effects of PA have been studied in both humans and model animals. Addition of PA at a PA/Zn molar ratio of 15:1 to a formulated diet caused a 50% reduction in Zn absorption in a group of young men (Turnlund et al., 1984). Another study reported a decrease of 82% in Fe absorption upon dietary PA supplementation at a PA/Fe molar ratio of ∼5:1 (Hallberg et al., 1989). Although it is difficult to determine the lowest limit of a healthy dietary PA/Zn or PA/Fe ratio, mainly because of the interference from other dietary components, it is well documented that the negative effect of PA on the absorption of micronutrient ions is significant (Yu et al., 2010). In developing countries, where cereals serve as staple food, higher dietary PA poses an even greater threat to micronutrient sufficiency than in developed countries (Ma et al., 2007; Al Hasan et al., 2016). Excreted PA from monogastric livestock pollutes the environment. Because monogastric animals (poultry and swine) cannot metabolize PA, excreted PA in the feces contributes to freshwater eutrophication (Brinch-Pedersen et al., 2002). Breeding for low PA crop varieties offers durable solution to these concerns.

Grains from low phytic acid (*lpa*) mutants contain significantly lower PA than the wildtype in maize (Raboy et al., 2000; Shi et al., 2003; Shi et al., 2005). Nutritional benefits of *lpa* were demonstrated in an animal experiment where rats fed with *lpa* maize meal absorbed twice the Zn as compared to those fed normal meal (Lonnerdal et al., 2011). But undesirable pleiotropic effects, for example, reduced seed germination and low grain yield, associated with the *lpa* mutant have limited its breeding application (Raboy et al., 2000; Pilu et al., 2005). Alternatively, a number of transgenic approaches silencing different steps of the PA biosynthetic pathway resulted in lower grain PA content (Kuwano et al., 2006; Shi et al., 2007; Ali et al., 2013). However, consumer opposition to genetically modified crops makes it an uphill task for these products to be made available for the target geographies, particularly when stewardship remains a concern (Wulff and Dhugga, 2018). It is desirable, thus, to explore genetic determinants of seed PA concentration, so that its content in the grain could be reduced within the constraints of natural variation by forward breeding.

Recently, a genome-wide association study (GWAS) was reported on exploring the genetics of the PA content in the rice grain (Perera et al., 2019). With only 69 accessions and inconsistent planting conditions (field and glasshouse), the results were inconclusive. Clearly, the throughput of PA quantification has been an impediment in studying the genetics of its accumulation in the grain (Schlemmer et al., 2009; Sivakumaran and Kothalawala, 2018).

Currently, there are several PA quantification methods available (Gao et al., 2007; Sivakumaran and Kothalawala, 2018). A commonly used method involves forming a complex of PA in the extracted sample with ferric salts, centrifugation to remove the precipitate, and then measuring residual ferric in the supernatant colorimetrically after mixing with sulfosalicylic acid, which forms a colored complex with ferric ions (Gao et al., 2007). Alternatively, the precipitate can be resuspended and further analyzed to directly measure PA. Other methods involve high performance liquid chromatography, ion exchange chromatography, or ^31^P nuclear magnetic resonance. These low throughput methods are not suitable for screening large breeding populations.

In this report we present an enzyme-base colorimetric method for high-throughput PA quantification. We used this method to measure the grain PA content of more than 300 field-grown wheat lines over 3 years. GWAS revealed several markers significantly associated with the PA content. Selection for as few as two of these markers could significantly reduce the grain PA content. Our study paves the way to breed for low grain PA by direct forward selection in breeding populations, at least in the later stages of a breeding program.

## Results and Discussion

### Development of High-Throughput Assay for Phytic Acid Quantification

**FIGURE 1:**
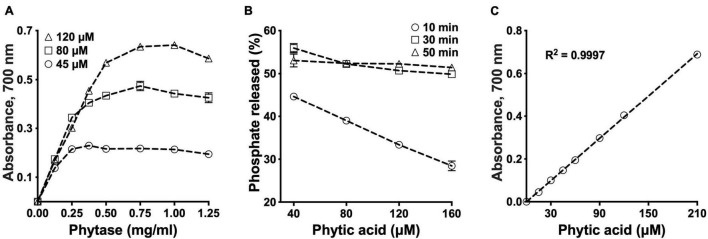
Standardization of phytic acid (PA) hydrolysis conditions. Optimization of the phytase concentration in PA hydrolysis **(A)**. PA was treated with phytase in a series of concentrations for 30 min at 55^°^C. Optimization of the reaction time for PA hydrolysis **(B)**. PA was treated with 0.75 mg/ml phytase at 55^°^C. PA standard curve **(C)**. All values are means over two replications.

We used a commercially available phytase, an enzyme that cleaves phosphate groups from PA, to develop a high-throughput assay for PA determination. Phosphate release peaked at a phytase concentration of 0.75 mg/ml (Figure 1A). At 55^°^C, maximal amount of phosphate was released after 30 min of incubation (Figure 1B). No more than 52% of the total expected phosphate could be released regardless of the initial amount of PA in the reaction, the incubation time and the amount of enzyme used (Figure 1B and Supplementary Figure 1). Absorbance (OD_700_) was linear over a range of 0–210 μM (*R*^2^ = 1), which is equivalent to 0–16.8 nmol or 0–11 μg PA in each 100 μl reaction (80 μl PA standard + 20 μl phytase, Figure 1C). Despite minor variation for the background phosphate and the phytase protein in different batches of the commercial enzyme, no differences in enzyme activity were observed as long as the protein concentration was adjusted to 0.75 mg/ml in the reaction (Supplementary Figure 2). The phytase solution was stable for up to 6 weeks at 4^°^C after the initial preparation (Supplementary Figure 3).

We did not detect any additional release of phosphate by alkaline phosphatase after the phytase treatment (Supplementary Figure 4). In contrast, McKie and McCleary (2016) reported sequential hydrolysis using phytase and then alkaline phosphatase released all the phosphate from PA. Phytase from wheat bran used in our assay is classified as a 4-phytase (1D-numbering system, enzyme commission number 3.1.3.26), which first hydrolyzes the phosphate group at the D-4 position of the *myo*-inositol ring, resulting in *myo*-inositol 1,2,3,5,6-pentakisphosphate (Konietzny and Greiner, 2002). Then, a series of lower *myo*-inositol phosphates are produced, with *myo*-inositol triphosphate as the main product, which is in accordance with the 50% PA hydrolysis rate we observed (Tomlinson and Ballou, 1962). However, lower *myo*-inositol phosphates with three or fewer phosphate residues cannot be hydrolyzed by most plant alkaline phosphatases (Konietzny and Greiner, 2002). Alkaline phosphatase used in our study from calf intestine (enzyme commission number 3.1.3.1) is capable of hydrolyzing *myo*-inositol monophosphate (*m*IMP). However, given the extremely low, if any, occurrence of *m*IMP in the PA hydrolysis products after phytase treatment, further release of phosphate was unlikely (Tomlinson and Ballou, 1962). In addition, phosphate, produced during each step of PA hydrolysis (averaged at 0.2 mM in our experiment) can strongly inhibit alkaline phosphatase (*K*_*i*_ = 0.03 mM) (Kaufman and Kleinberg, 1975). Unlike the wheat bran phytase we used in our study, other phytases, for example fungal phytases, can cleave a larger number of phosphate groups from the PA (Rao et al., 2009). The difference between our results and those of McKie and McCleary (2016) could possibly be attributed to the different phytases used in each study. As long as the phytase enzyme consistently cleaves the same number of phosphate groups from PA in different samples, PA quantification would be consistent.

Free phosphate concentration in wheat seed extracts was negligible as compared to that released by phytase hydrolysis (Supplementary Figure 5A). A comparison of the PA content measured from 330 wheat lines with or without correcting for free phosphate suggested that it was unnecessary to measure the background phosphate for GWAS (Supplementary Figure 5B), which would help reduce the number of assays by half.

A single-enzyme based, high-throughput assay we report here is reliable in quantifying grain PA content (see Supplementary Figure 6. Schematic Diagram of High-throughput Phytic Acid Quantification Method). We compared our method with a commercially available, low-throughput assay and obtained similar results (*R*^2^ = 0.97, Supplementary Figure 7; McKie and McCleary, 2016).

### Variation for Phytic Acid in Wheat Genotypes

**FIGURE 2:**
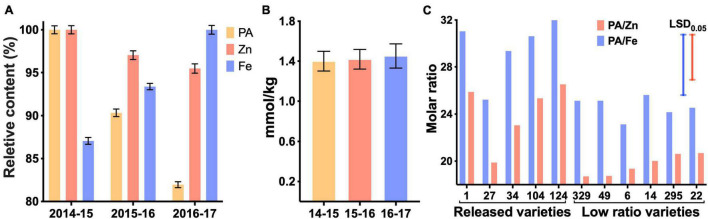
Variations for phytic acid (PA), Fe and Zn over three growing seasons **(A,B)** and selected lines differing for PA to Fe and Zn molar ratios **(C)**. Numbers on the *X*-axis refer to years 2014–2017. Relative values of PA, Zn, and Fe **(A)**. Maximum concentrations were 1.72% for PA in 2014–2015, 38.1 mg/kg for Fe in 2016–2017 and 52.7 mg/kg for Zn in 2014–2015. Total metal ion concentration in each growing season **(B)**. Only the released wheat varieties and selected varieties with low PA/Zn and PA/Fe were plotted **(C)**. Blue and red bars represent the least significant difference. Numbers on the *X*-axis correspond to the breeding lines.

We measured grain PA in the wheat lines from the HarvestPlus Association Mapping (HPAM) population grown at CIMMYT over 3 years (Velu et al., 2016, 2018). The PA content ranged from 1.06 to 1.72% (w/w) in 2014–2015, from 0.95 to 1.51% in 2015–2016 and from 0.90 to 1.35% in 2016–2017 (Supplementary Figure 8). The mean PA content was highest in the 2014–2015 season and lowest in the 2016–2017 season, indicating a role of environmental factors in PA accumulation (Figure 2A). Zinc content also followed the same pattern as PA but iron content exhibited an opposite pattern. Whereas it is difficult to pinpoint the environmental factors underlying the observed variation (Supplementary Figure 9), the data suggest that divalent cations, at least iron and zinc together, maintain a homeostatic molar concentration in the grain in fluctuating environments (Figure 2B).

The HPAM panel used in our study was previously used in the CIMMYT biofortification breeding program, the aim of which was to breed lines with increased grain Fe and Zn contents (Velu et al., 2016, 2018). Several biofortified varieties have been released from this program in India (WB-02, PBW01Zn, and HUW711), Pakistan (Zincol-16 and Akbar-19), Bangladesh (BARI-Gom 33) and Nepal (Zinc Gahun 1, Zinc Gahun 2, Bheri-Ganga, Himganga, and Khumal-Shakti) (Singh et al., 2017). However, as important factors in determining the bioavailability of Fe and Zn, the molar ratios of PA to Fe and Zn were not available at the time these selections were made. As a result, some of the released varieties with higher grain Fe and Zn did not have an optimal PA/Zn or PA/Fe molar ratios (Figure 2C).

**FIGURE 3:**
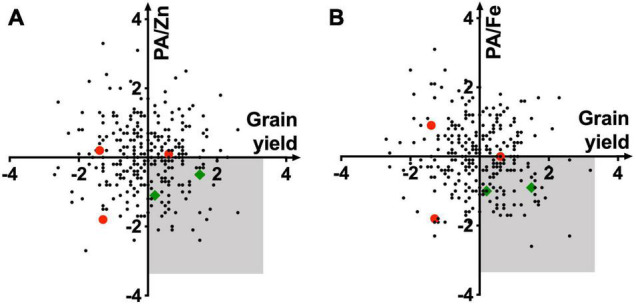
Relationship of standardized PA/Zn **(A)** and PA/Fe **(B)** molar ratios with grain yield. Quadrangles representing wheat lines with high bioavailability of Zn and Fe and high grain yield are shaded in gray. Red dots and green diamonds represent wheat lines released as commercial varieties. PA, Fe, Zn, and yield data were averaged over 2015–2016 and 2016–2017 seasons.

Plotting of standardized variables of PA/Fe and PA/Zn ratios against grain yield allowed identification of high yielding lines with relatively low grain PA (Figure 3). A lack of correlation (*R*^2^ = 0.01) between these two traits makes it possible to advance the recombinants with high yield and low grain PA. Varieties in the bottom-right quadrant (shaded area) should be prioritized during selection for their high bioavailability of Zn and Fe and grain yield. Among the five released breeding lines, only two met these criteria (Figure 3, green diamond). As other agronomic traits must be considered before varietal release, and the grain yield data used for this analysis were from small plots, our assay could be deployed later in the breeding cycle to advance those lines that have desired combinations of high grain metal ions and low PA.

### Genome-Wide Association Study for Phytic Acid Content

**FIGURE 4:**
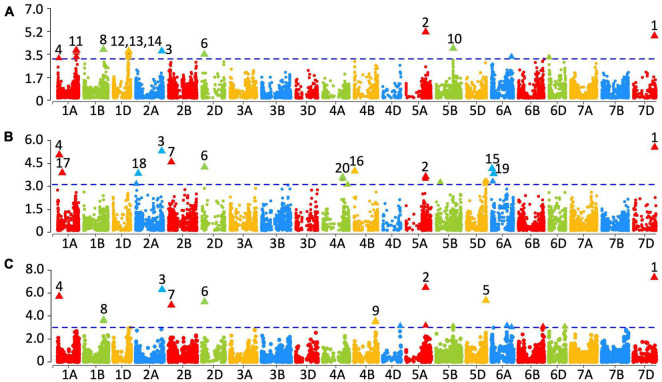
Markers significantly associated with grain phytic acid from year 2015–2016 **(A)**, 2016–2017 **(B)** and averaged over two years **(C)**. The –log_10_ *p*-values are shown on the *y*-axis. Markers that were significant at a *p*-value of 0.001 are numbered: (1) BobWhite_c34689_116, (2) IACX8282, (3) RFL_Contig3550_457, (4) BS00009885_51, (5) Excalibur_c48387_58, (6) BS00069899_51, (7) RAC875_rep_c109207_706, (8) RAC875_rep_c109215_398, (9) Kukri_c9545_864, (10) RAC875_c39141_55, (11) Excalibur_c1236_444, (12) Ra_c15730_3403, (13) Excalibur_c16570_925, (14) D_GDS7LZN02GSCOW_131, (15) RFL_Contig2815_1135, (16) BS00063809_51, 17) BS00048118_51, (18) Tdurum_contig48302_532, (19) BobWhite_c23888_124, (20) and Excalibur_rep_c70578_299.

Genome-wide association study for PA content was performed using the mixed linear model with the PA content evaluations in all 3 years and the best linear unbiased estimates (BLUEs) obtained from the two evaluations (2015–2016 and 2016–2017, Figure 4). The *p*-values for the significance of marker-trait associations, the additive effect of the markers on the PA content and the percentage variation in PA content explained by the markers are shown in Supplementary Table 1. In the 2015–2016 dataset, 23 markers were significantly associated with PA content at a *p*-value threshold of 0.001 (Figure 4A and Supplementary Table 1). Among those, the most significant marker, wsnp_BE444579B_Ta_2_1 on chromosome 3B (physical location unaligned in Refseq v1.0), followed by the marker IACX8282 on chromosome 5AL were also significant after Bonferroni correction (α level of 0.20). In addition, BS00009885_51 on chromosome 1AS, four markers on chromosome 1AL between base pair positions 529666321 and 530236376, RAC875_rep_c109215_398 on chromosome 1BL, eight markers on chromosome 1DL between 430565264 and 433276844 bps, RFL_Contig3550_457 on chromosome 2A, BS00069899_51 on chromosome 2DS, Ku_c69970_624 on chromosome 3AL, RAC875_c39141_55 on chromosome 5BL, IAAV5761 on chromosome 6AL, RAC875_c82406_177 on chromosome 6DS and BobWhite_c34689_116 on chromosome 7DL were also associated with PA content in the 2015–2016 dataset.

In the 2016–2017 dataset, 20 markers were significantly associated with PA content (Figure 4B and Supplementary Table 1). Among them, the most significant markers were BobWhite_c34689_116 on chromosome 7DL, RFL_Contig3550_457 on chromosome 2A and BS00009885_51 on chromosome 1A. Further-more, BS00048118_51 on chromosome 1AS, BS000716 30_51 and Tdurum_contig48302_532 on chromosome 2AS, RAC875_rep_c109207_706 on chromosome 2BS, BS00069 899_51 on chromosome 2DS, wsnp_BE444579B_Ta_2_1 on chromosome 3B (physical location unaligned in Refseq v1.0), Excalibur_rep_c70578_299 on chromosome 4AL, BS00063809_51 on chromosome 4BS, IACX8282 on chromosome 5AL, four markers on chromosome 5DL between 543272435 and 548368912 bps and three markers on chromosome 6AS including RFL_Contig2815_1135, Kukri_c10226_1815, and BobWhite_c23888_124 were also significantly associated with the PA content in the 2016–2017 dataset. No significant associations that were consistent with the 2015–2017 data sets were found in the 2014–2015 data set. It is possible that the environmental conditions that led to high grain PA in that year also contributed to the noise level, masking the genetic signal (Supplementary Figure 9).

In the dataset with the BLUEs obtained from the two evaluations, 17 markers were significantly associated with PA content (Figure 4C and Supplementary Table 1), among which wsnp_BE444579B_Ta_2_1on chromosome 3B (physical location unaligned in Refseq v1.0), BobWhite_c34689_116 on chromosome 7DL, IACX8282 on chromosome 5AL, RFL_Contig3550_457 on chromosome 2A, BS00009885_51 on chromosome 1A, Excalibur_c48387_58 on chromosome 5DL, BS00069899_51 on chromosome 2DS and RAC875_rep_c109207_706 on chromosome 2BS were also significant. In addition, RAC875_rep_c109215_398 on chromosome 1BL, IAAV2328 on chromosome 5AL, Kukri_c9545_864 on chromosome 4BL, IACX11246 on chromosome 4DL, IAAV2328 on chromosome 5AL, RAC875_c39141_55 on chromosome 5BL, Kukri_c1281_515 and IAAV5761 on chromosome 6AL, BS00067417_51 on chromosome 6BL, BS00109999_51 on chromosome 6DL and Kukri_c35508_426 on chromosome 7D were also significantly associated with PA content in the BLUEs dataset.

**FIGURE 5:**
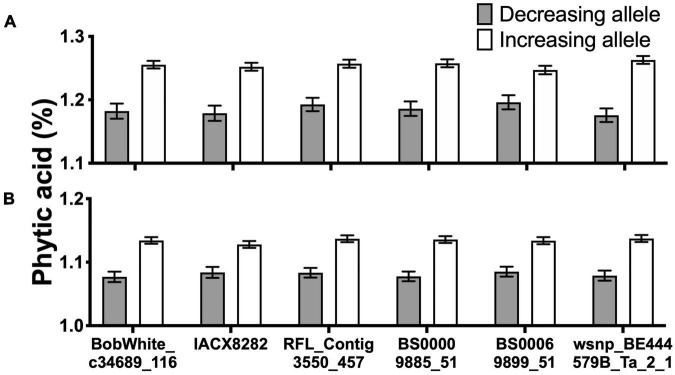
Grain phytic acid (PA) content in the presence or absence of desirable alleles. Six markers consistently associated with grain PA content in year 2015–2016 **(A)** and 2016–2017 **(B)**. All values are means over the respective number of lines for each allele.

**FIGURE 6:**
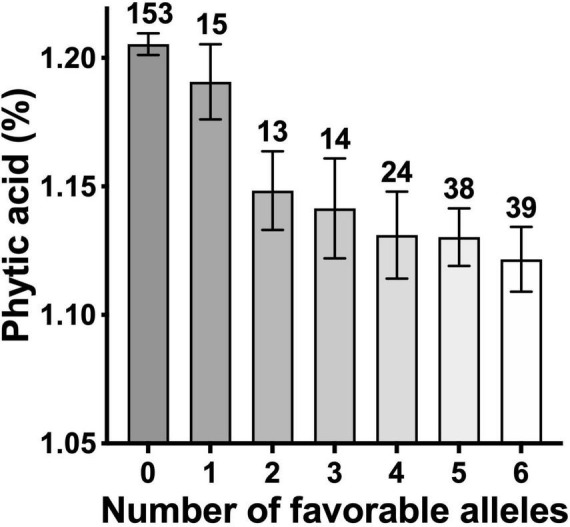
Phytic acid content in wheat lines as a function of number of favorable alleles. The number of lines in each group are shown on top of respective bars.

Overall, six consistent and significant associations for the PA content across two of the 3 years were obtained (Figure 5 and Supplementary Table 2). Significant phenotypic variation in PA content could be attributed to the alleles at these six markers (Figure 5). Of the 330 lines, 296 had the allelic fingerprints (increasing allele, decreasing allele, and heterozygote) of these six markers (Supplementary Figure 10). Approximately half the lines, 153, had no favorable allele (less PA), 104 lines had 1–5, and 39 lines had all six 6 favorable alleles (Supplementary Table 2). The effect of four of the six favorable alleles on grain PA was additive, showing a reduction from 1.21% with no favorable allele down to 1.13% (Figure 6), which could potentially increase the bioavailability of Fe and Zn by ∼7%. These results highlight the value of PA assays in lowering its content through forward selection.

Genes within 1 Mbp upstream and downstream of the six markers are listed in Supplementary Table 3. An ABC-2 type transporter gene (TraesCS5A01G344500) on chromosome 5A, which is located ∼0.42 Mbp from marker IACX8282, presents a candidate for genetic manipulation. A similar gene has been previously shown to control the PA level in the maize grain and soybean seed (Shi et al., 2007). Silencing this gene specifically in the embryo caused a 75% reduction in the seed PA content without any noticeable pleiotropic effects (Shi et al., 2007). Two other genes (TraesCS7D01G454300 and TraesCS7D01G454500) annotated as dolichyl-diphosphooligosaccharide-protein glycosyltransferase were respectively located 1.3 and 130 Kbp distal to marker BobWhite_c34689_116 on chromosome 7D. These genes were upregulated in low PA soybean mutants, suggesting their possible role in PA formation (Yuan et al., 2017). All these genes can be silenced by gene editing. Knocking out the ABC transporter gene in particular might lead to a greater reduction in grain PA than the allelic variant in our study with the assumption that the desirable allelic variant has reduced function.

The value of our assay in screening breeding populations for improved biofortification can be judged from a reevaluation of the original selections that were made based only on high grain mineral ion concentrations (Velu et al., 2016, 2018). Fewer than 1/3rd of the 330 lines contained four or more desirable alleles for low grain PA (Figure 6), the frequency that is also reflected in the commercial varieties that have been released in different countries (Figure 2C). Only one of the five released varieties had desirable PA to mineral ion ratios (Figure 2C). In the future, aside from their usefulness in direct selection for low grain PA, the desirable haplotypes could be used to prescreen the parental lines to set up crosses in the breeding programs targeted toward improving grain biofortification (Figures 4–6).

We have presented a high-throughput, reliable and easy-to-use PA quantification method that is scalable for phenotyping large breeding populations. Markers identified in our wheat grain PA GWAS could assist in screening late-stage breeding populations for the identification of low grain PA wheat varieties. Further, our method is widely applicable to other crops.

## Experimental Procedures

### Wheat Population and Field Experiment

The HarvestPlus Association Mapping (HPAM) panel consisted of 330 wheat lines from CIMMYT’s biofortification breeding program. This genetically diverse panel could be divided into five sub-populations that were derived from: (1) landraces; (2) *Triticum durum*-based synthetic hexaploid derivatives; (3) *Triticum dicoccon*-based synthetic hexaploid derivatives; (4) *Triticum spelta* derivatives; and (5) CIMMYT’s pre-breeding derivatives of diverse progenitors including *Triticum polonicum* (Velu et al., 2018). The panel was grown in a randomized complete block design in two replications with a plot size of 2 m^2^ at CIMMYT’s experimental station in Ciudad Obregon, Sonora, Mexico (27°24′N, 109°56′W) during three successive crop seasons (2014–15, 2015–16, and 2016–17). Trials were irrigated five times throughout the crop cycle and fertilized at a rate of 200:50 (N:P) kg.ha^–1^, of which 50:50 was applied in pre-sowing and 150:00 at tillering stage. Diseases and pests were controlled chemically, whereas weeds were controlled manually and chemically according to CIMMYT’s standard protocols. Zinc was applied at a rate of 25 kg.ha^–1^ as ZnSO_4_.7H_2_O over three crop cycles to reduce soil heterogeneity for this micronutrient. Soil analysis of the experimental area showed an average Zn concentration of 1.2 ppm at soil depth of 0–30 cm, and 0.86 ppm at a soil depth of 30–60 cm. The average Fe concentration in the soil was 5.0 and 6.1 ppm, at 0–30 and 30–60 cm soil depth, respectively. Rest of the details were as previously reported (Cu et al., 2020).

Wheat head samples were collected from five plants per genotype in each plot at physiological maturity in each of the seasons. All samples were oven dried at 40°C for 4 days, threshed, grain separated and subjected to subsequent analyses. Iron and zinc were determined as previously described (Cu et al., 2020).

### Phytic Acid Extraction From Wheat Seeds

Wheat grains (10 grains per line) were dried at 60^°^C for 3 days and ground using a GenoGrinder (model 2010, SPEX Sample Prep, United States) with small grinding vial set (6751, SPEX Sample Prep) for 15 s four times at 1750 rpm. Fifty mg of each flour sample were weighted into individual microtiter tube in a 96-tube rack format, 600 μl of 0.6 M HCl added using a Liquidator96 96-well pipettor (LIQ-96-200, METTLER TOLEDO, United States). After sealing the tubes, PA was extracted at room temperature on a shaker at 150 rpm overnight. The sample racks were centrifuged at 4800 g for 10 min in a Megafuge 40R centrifuge (Thermo Scientific, United States, rotor 75003607), followed by transferring 50 μl supernatant into new microtiter tubes. The clear PA extracts were neutralized by mixing with 50 μl 0.6 M NaOH and then diluted with 900 μl 0.2 M sodium acetate (NaOAc), pH 5.5.

### Phytase Working Solution

Phytase (P1259, Sigma-Aldrich, United States) was dissolved in 0.2 M NaOAc, pH 5.5, to give a concentration of 10 mg/ml. Actual phytase concentration was quantified using a bicinchoninic acid protein determination kit (BCA1 and B9643, Sigma-Aldrich, United States) and a microplate reader (FLUOstar Omega, BMG Labtech, Germany). The phytase working solution was prepared by adjusting the actual phytase concentration to 4 mg/ml using the NaOAc buffer.

### Phytic Acid and Phosphate Standards

Phytic Acid solution (593648, Sigma-Aldrich, United States) was used to prepare a series of PA standard solutions in the NaOAc buffer. Phosphate standards were prepared by dissolving KH_2_PO_4_ in the NaOAc buffer.

### Phytic Acid Hydrolysis and Quantification

High-throughput assays were performed essentially as described for other traits previously (Silva et al., 2017, 2018). PA in 80 μl diluted extract (and PA standards and phosphate standards) was hydrolyzed by mixing with 20 μl phytase working solution and incubated at 55^°^C for 30 min. Then each reaction was mixed with the 100 μl color reagent and incubated at 37^°^C for 30 min. The color reagent is consisted of the four solutions in a ration of A:B:C:D = 1:1:1:2 (A. 2.5% (w/w) (NH_4_)_2_MoO_4_, B. 3 M H_2_SO_4_, C. 10% (w/w) ascorbic acid and D. H_2_O). Then the reactions were centrifuged at 4700 rpm for 10 min. 100 μl supernatant of each reaction was transferred to a 96-well plate and absorbance was measured at a wavelength of 700 nm. The wavelength was determined by scanning through the absorbance of phosphate standards.

### Genotyping Data, Population Structure, and Kinship Analysis

The 330 lines in the HPAM panel were genotyped using the Illumina iSelect 90 K Infinitum single nucleotide polymorphism array. Quality control of the genotyping data was done by filtering markers with greater than 70% missing data, less than 5% minor allele frequency and greater than 10% heterozygosity. We then obtained the physical positions of the markers in the reference genome of wheat (Refseq v1.0) available at https://triticeaetoolbox.org/wheat/maps (Appels et al., 2018). Plot of SNP densities in different chromosomes or the number of SNPs within a 10 Mb window revealed moderate to good coverage in the telomeric regions, and a relatively lower coverage in the centromeric regions (Supplementary Figure 11). The highest number of markers were in the B genome (4,323 markers, 42.6%), followed by the A genome (4,038 markers, 39.8%) and the D genome (1,797 markers, 17.7%).

Population structure of the lines in the panel was done using the principal component analysis in TASSEL (Trait Analysis by aSSociation Evolution and Linkage) version 5. Moderate population structure was observed and the first two principal components explained 10.9 and 7.2% of the variation, respectively (Supplementary Figure 12). In addition, we also obtained the kinship between the lines using the centered identity-by-state method in TASSEL version 5 (Endelman and Jannink, 2012). Several lines formed clusters with a relationship of 0.4–0.6, while only a few lines had a relationship of over 0.7 (Supplementary Figure 13).

### Genome-Wide Association Mapping and Allelic Fingerprinting

We performed genome-wide association mapping for PA content in TASSEL version 5 with the mixed linear model and used the optimum level of compression and the “population parameters previously determined” options (Yu et al., 2006; Zhang et al., 2010). The first two principal components that accounted for the population structure were used as fixed effects and the kinship matrix among the lines was used as the random effect in the mixed linear model (Price et al., 2006). We obtained the marker *p*-values, additive effects and percentage variation explained by each marker and then plotted the Manhattan plots with the - log_10_ *p*-values of the markers using the “R” package CMplot (Lilin-Yin, 2018). To declare significance of the markers, we used a *p*-value threshold of 0.001 and also corrected for multiple testing using the Bonferroni method at an α level of 0.20. The markers that were consistently associated with PA content in the two evaluations were then identified and the alleles associated with increasing and decreasing the PA content at these markers were fingerprinted in all the lines. We then determined the relationships between the number of PA favorable alleles (PA decreasing alleles) at the consistent markers and the PA content and visualized them using box plots, created using the R package, “ggplot2” (Ginestet, 2011). Finally, we also obtained the genes that were 100 Mb upstream and downstream of the consistently significant markers using Jbrowse available at https://wheat-urgi.versailles.inra.fr/Tools/JBrowse and explored the genes in the interval to identify potential PA-associated candidate genes.

## Data Availability Statement

The datasets presented in this study can be found in the Supplementary Material.

## Author Contributions

ZW, HSD, and KSD developed and refined the assay. ZW oversaw screening of the grain samples. MP, UIM, and AA carried out the assays in the population and MII compared different methods. VG and RPS conducted the field experiments and provided the grain samples. PJ carried out GWAS analysis. ZW and KSD wrote and revised the manuscript. All authors contributed to the article and approved the submitted version.

## Conflict of Interest

The authors declare that the research was conducted in the absence of any commercial or financial relationships that could be construed as a potential conflict of interest.

## Publisher’s Note

All claims expressed in this article are solely those of the authors and do not necessarily represent those of their affiliated organizations, or those of the publisher, the editors and the reviewers. Any product that may be evaluated in this article, or claim that may be made by its manufacturer, is not guaranteed or endorsed by the publisher.
