# Supplementary material for: Genome-Wide Association Study of Phytic Acid in Wheat Grain Unravels Markers for Improving Biofortification
Source: Front Plant Sci. 2022 Feb 15;13:830147. doi: 10.3389/fpls.2022.830147 (PMC8886111; doi:10.3389/fpls.2022.830147)
Supplement: Supplementary file 1 [file Data_Sheet_1.PDF]

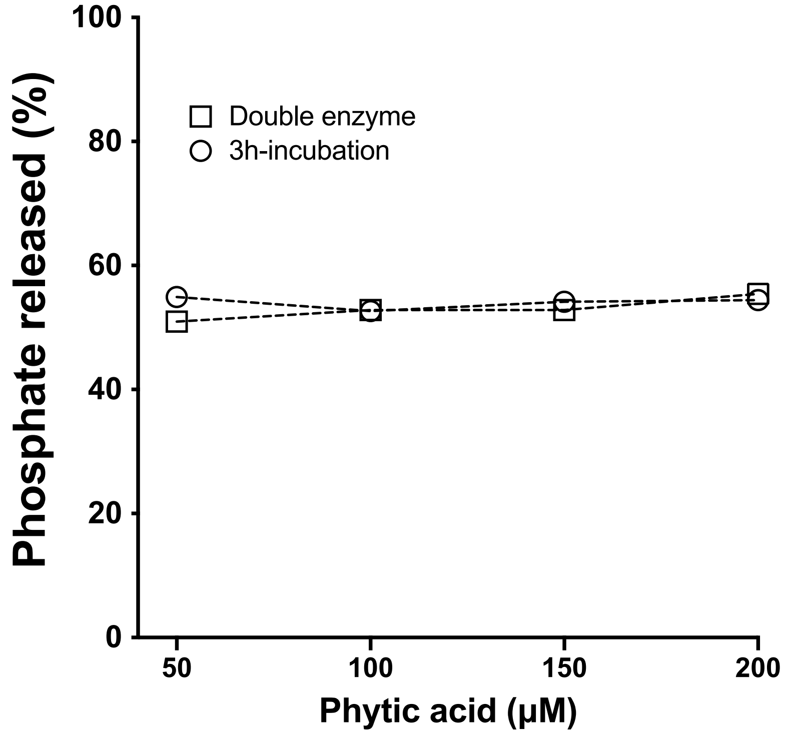

Supplementary Figure 1. Standardization of phytic acid (PA) hydrolysis conditions.
PA was hydrolysed with 0.75 or 1.5 mg/ml phytase for 3 h at 55 °C. Values are means over two replications.

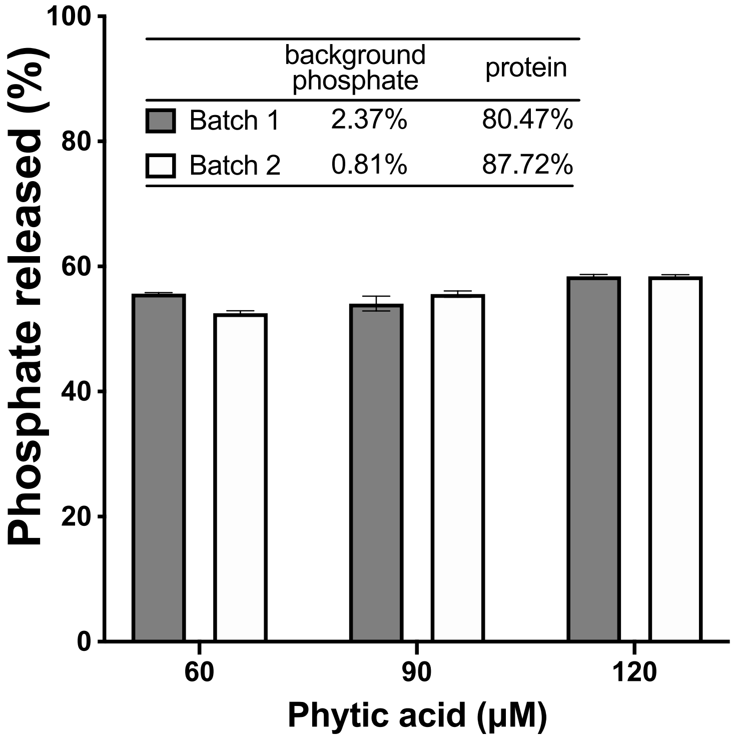

Supplementary Figure 2. Phytase activity in different batches from the supplier. Phytic acid was hydrolyzed using 0.75 mg/ml phytase.
Background phosphate concentration and protein concentration of the two phytases were normalized before the reaction and are listed in the inset table. Values are means over two replications.

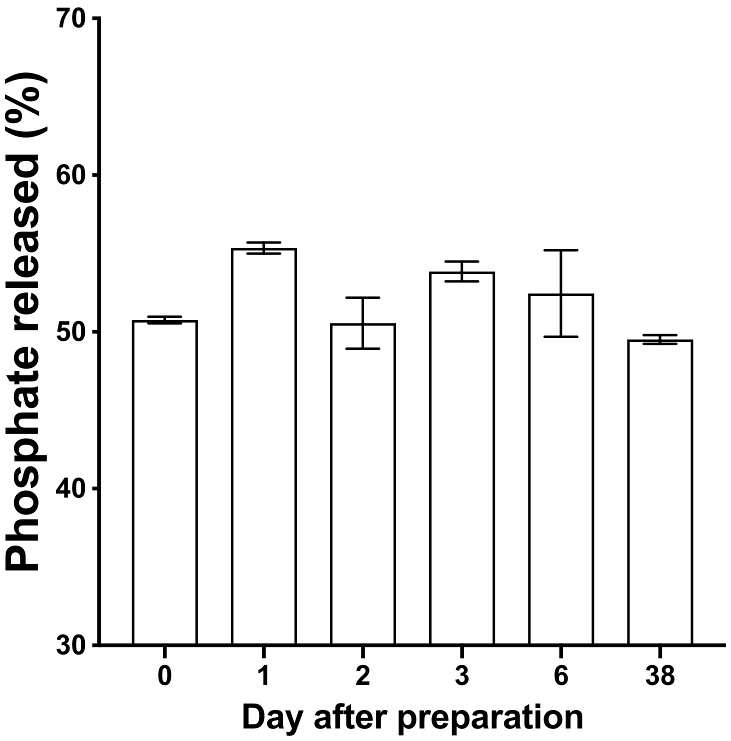

Supplementary Figure 3. Phytase shelf life at 4 ^{o}C. Phytase activity was measured on different days after initial enzyme preparation. Values are means over two replications.

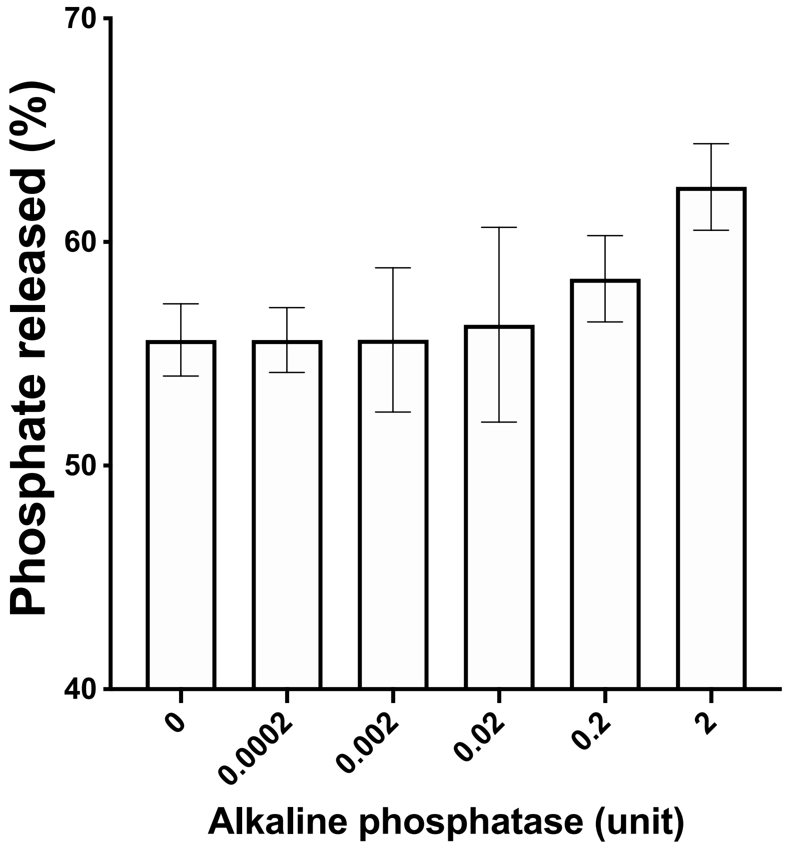

Supplementary Figure 4. Hydrolysis using alkaline phosphatase.
Phytase-treated phytic acid standards were further treated with alkaline phosphatase at pH 9.8 for 30 min at 37 ^{o}C. Values are means over two replications.

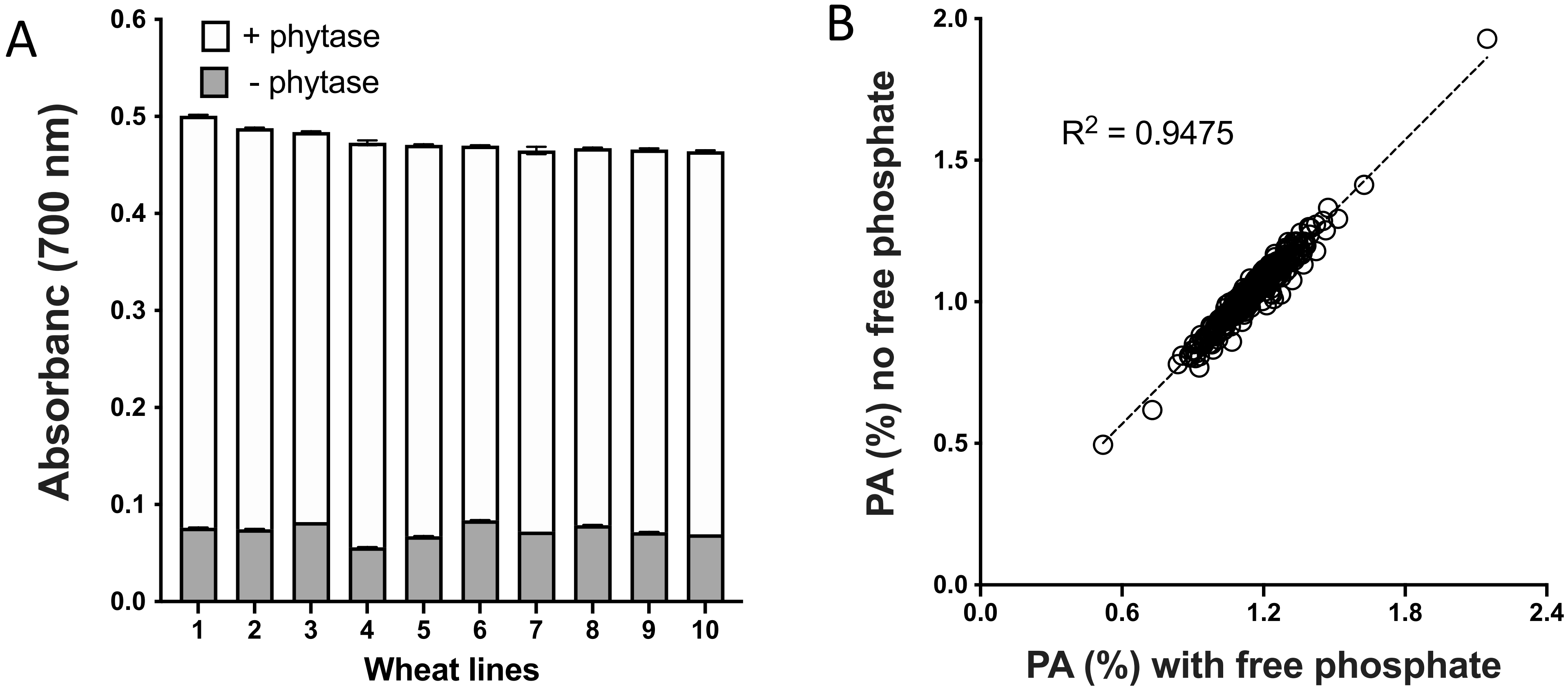

Supplementary Figure 5. Phytic acid (PA) quantification in wheat grains.
PA from ten randomly selected wheat lines was measured with or without phytase treatment. Absorbance from free phosphate in wheat grains is shown in grey (A). Values are means over two replications. Correlation of PA content from 307 wheat lines measured with or without subtracting free phosphate (B) .

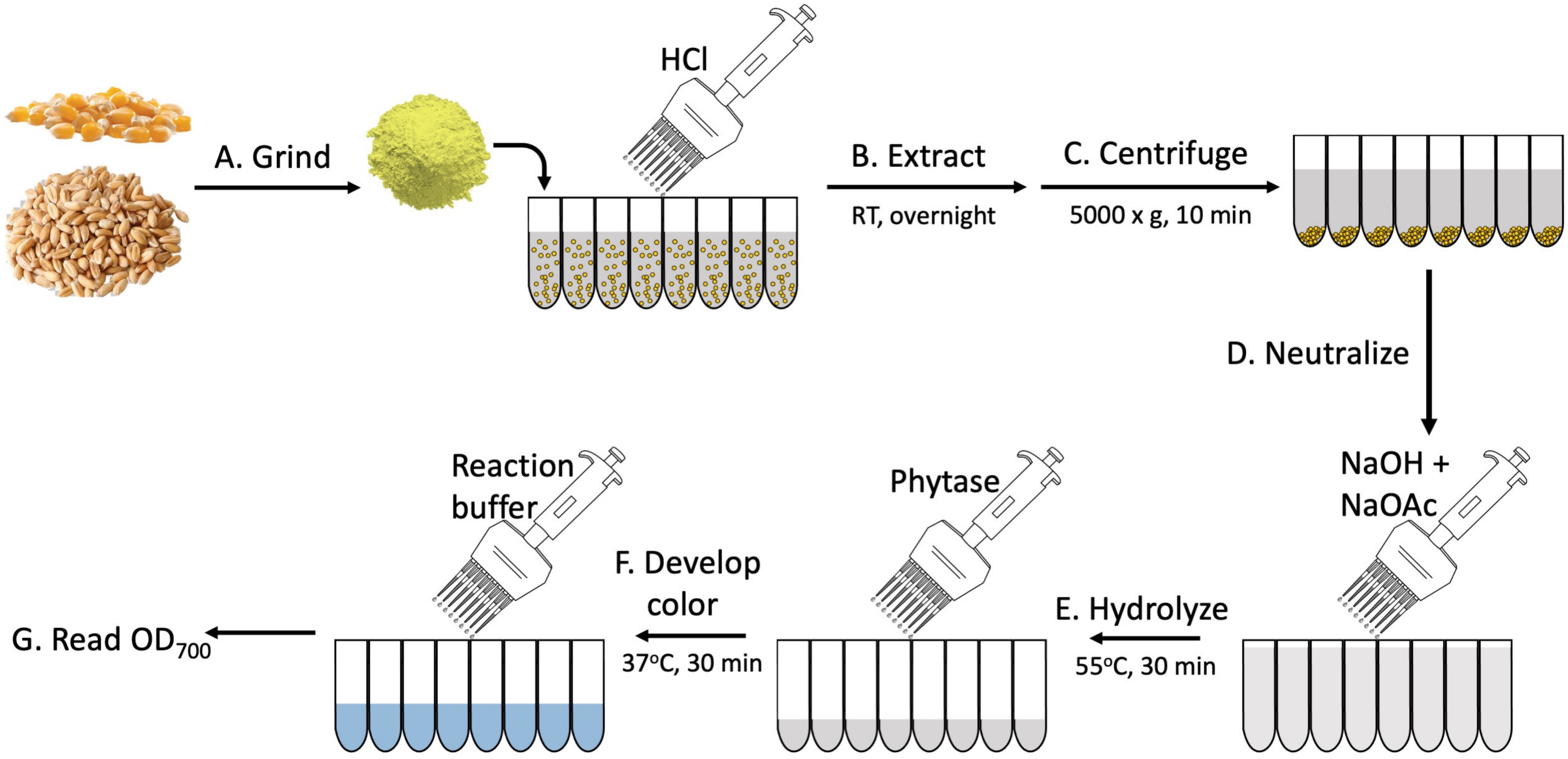

Supplementary Figure 6. Schematic diagram of the high-throughput phytic acid quantification.

Seeds were ground in GenoGrinder (A), 50 mg flour mixed with 600 µl 0.6 M HCl and placed on a rotary shaker overnight at room temperature (B). Following centrifugation (C), 50 µl extract was mixed with 50 µl 0.6 M NaOH and 900 µl 0.2 M sodium acetate (NaOAc) buffer, pH 5.5 (D). Then 80 µl diluted extract was mixed with 20 µl phytase solution (4 mg/ml) to hydrolyze phytic acid (E). Finally, the released phosphate was measured colorimetrically using a plate reader (F and G).

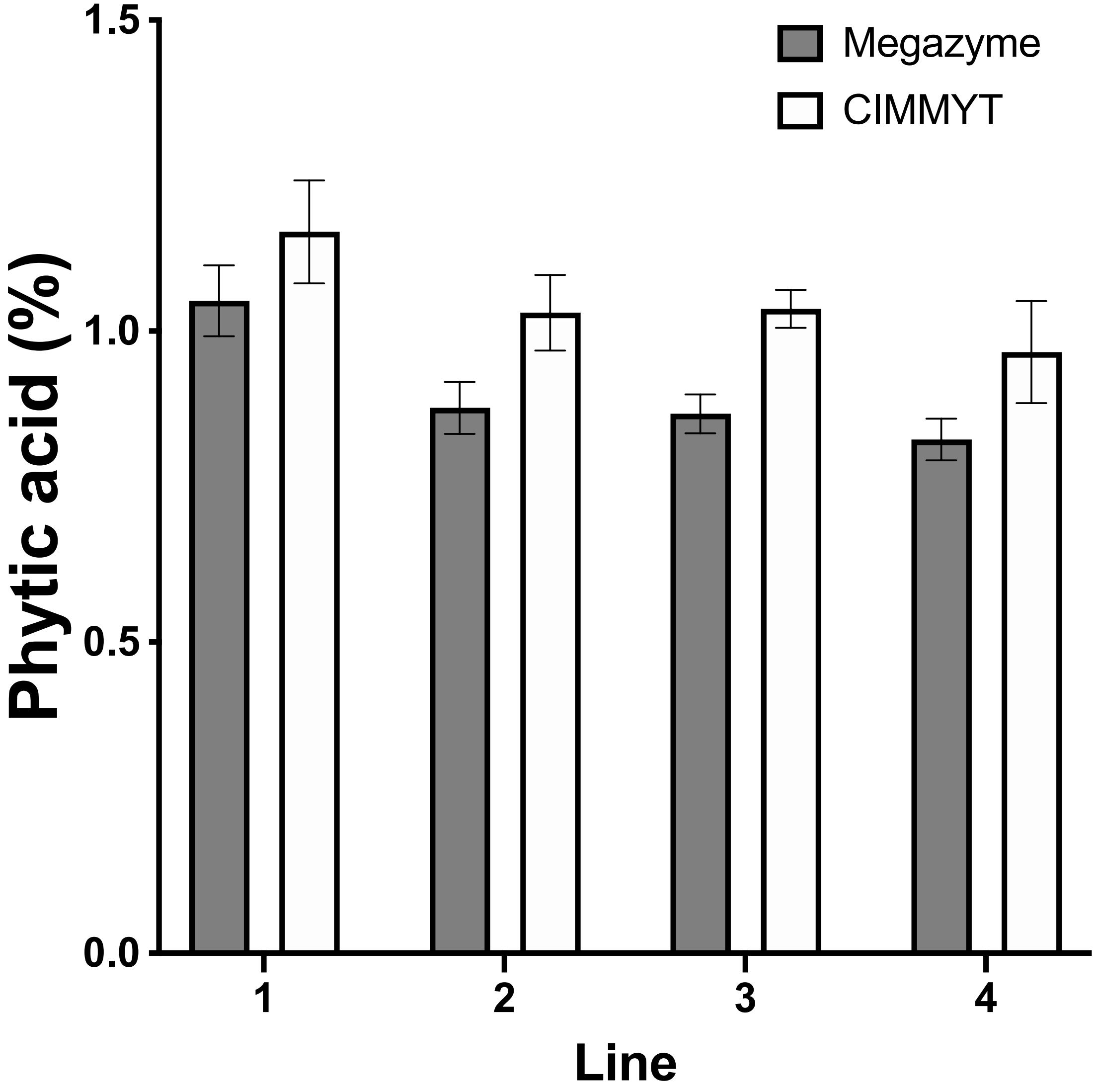

Supplementary Figure 7. Comparison between the Megazyme kit and single-enzyme phytic acid (PA) quantification method.

PA content was determined from four randomly selected lines using the two methods. Values are means over eight replications.

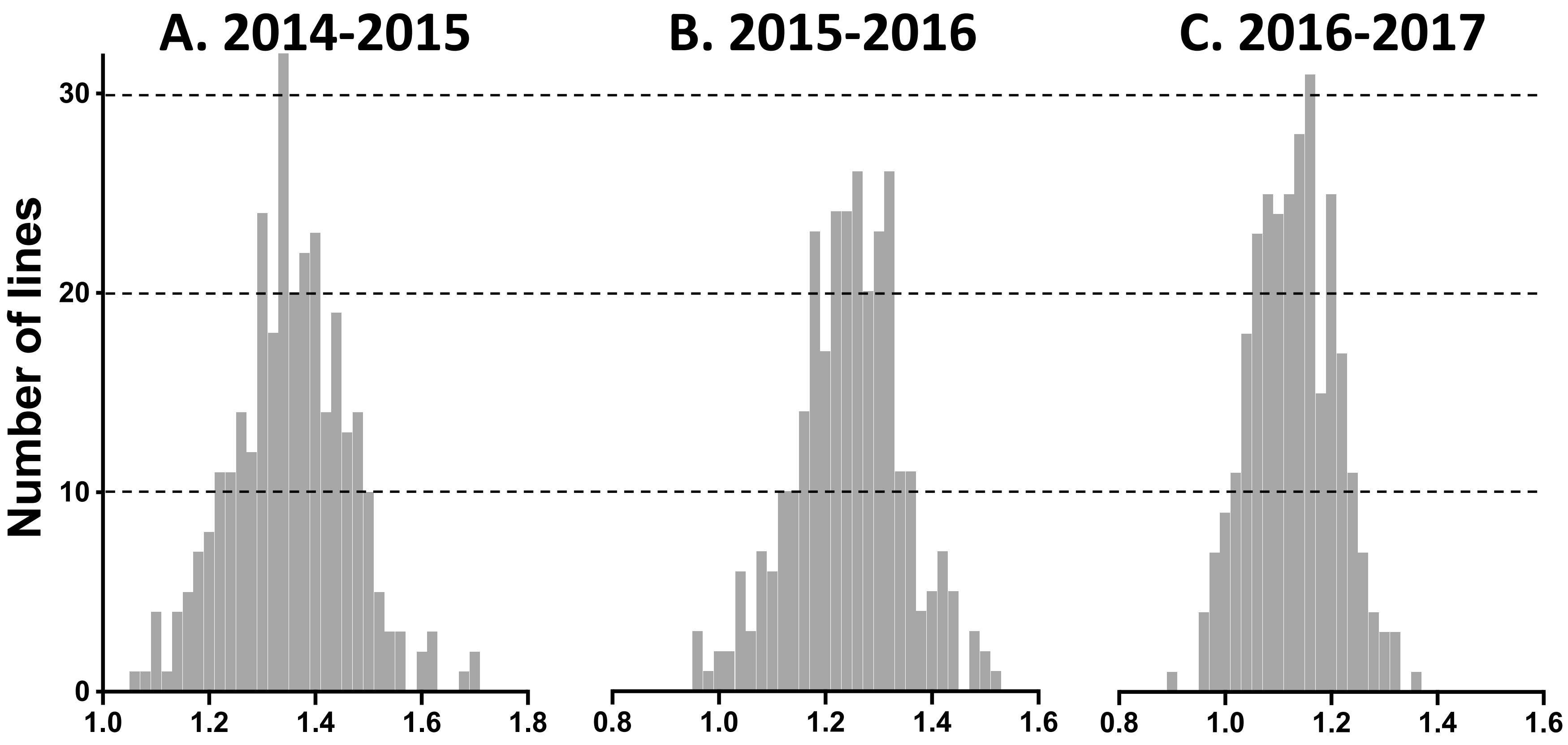

Supplementary Figure 8. Distribution of PA content in 330 wheat lines in 2014-2015 (A), 2015-2016 (B) and 2016-2017 growing seasons (C).

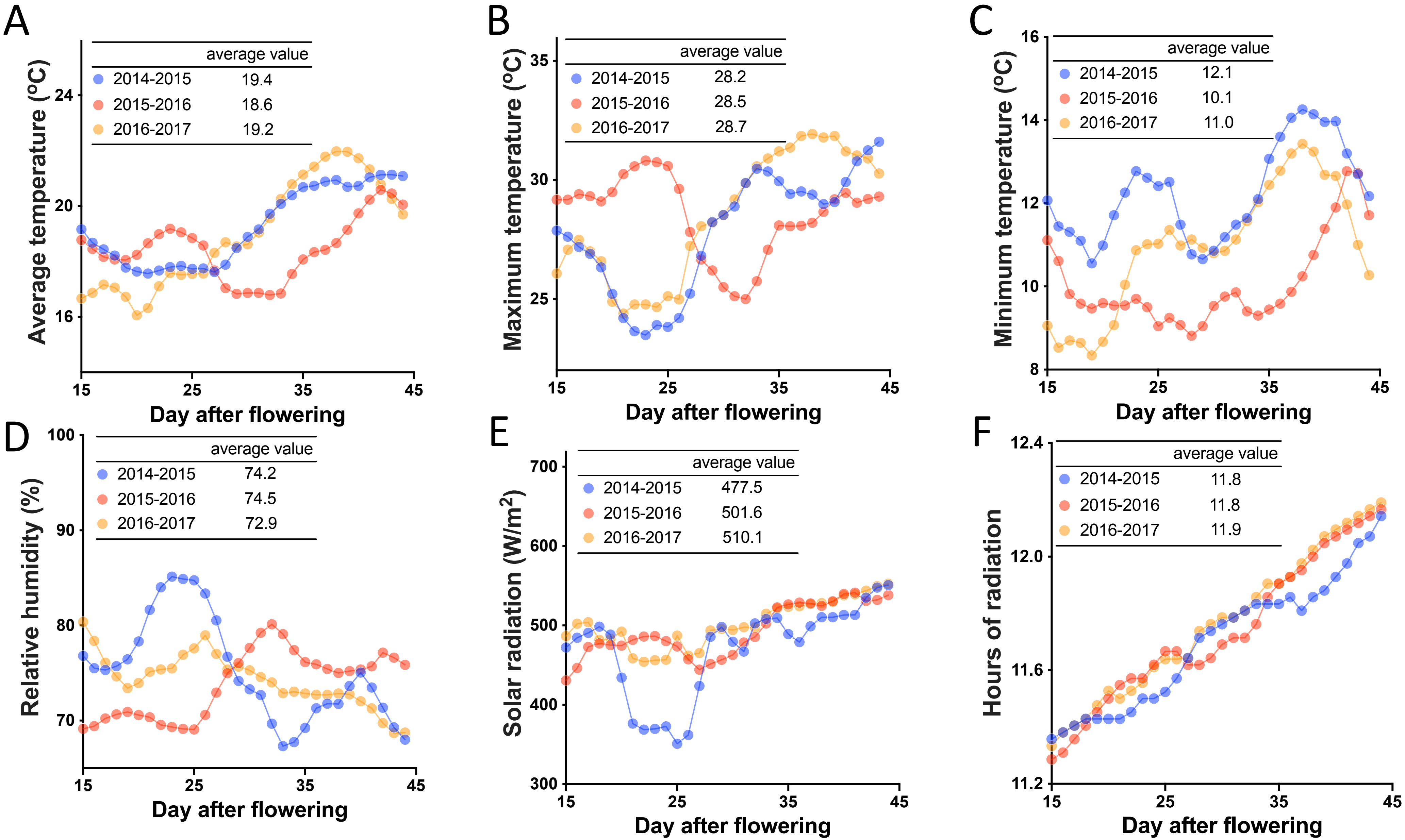

Supplementary Figure 9. Environmental variables during grain filling stage in Obregon, Mexico.
(A) Average temperature, (B) maximum temperature, (C) minimum temperature, (D) relative humidity, (E) solar radiation and (F) radiation hours. Moving averages over 7 days were plotted starting at 15 days after flowering. Data were provided by CIMMYT Oregon station. Average PA concentration was 1.36% in 2014-2015, 1.23% in 2015-2016 and 1.12% in 2016 to 2017.

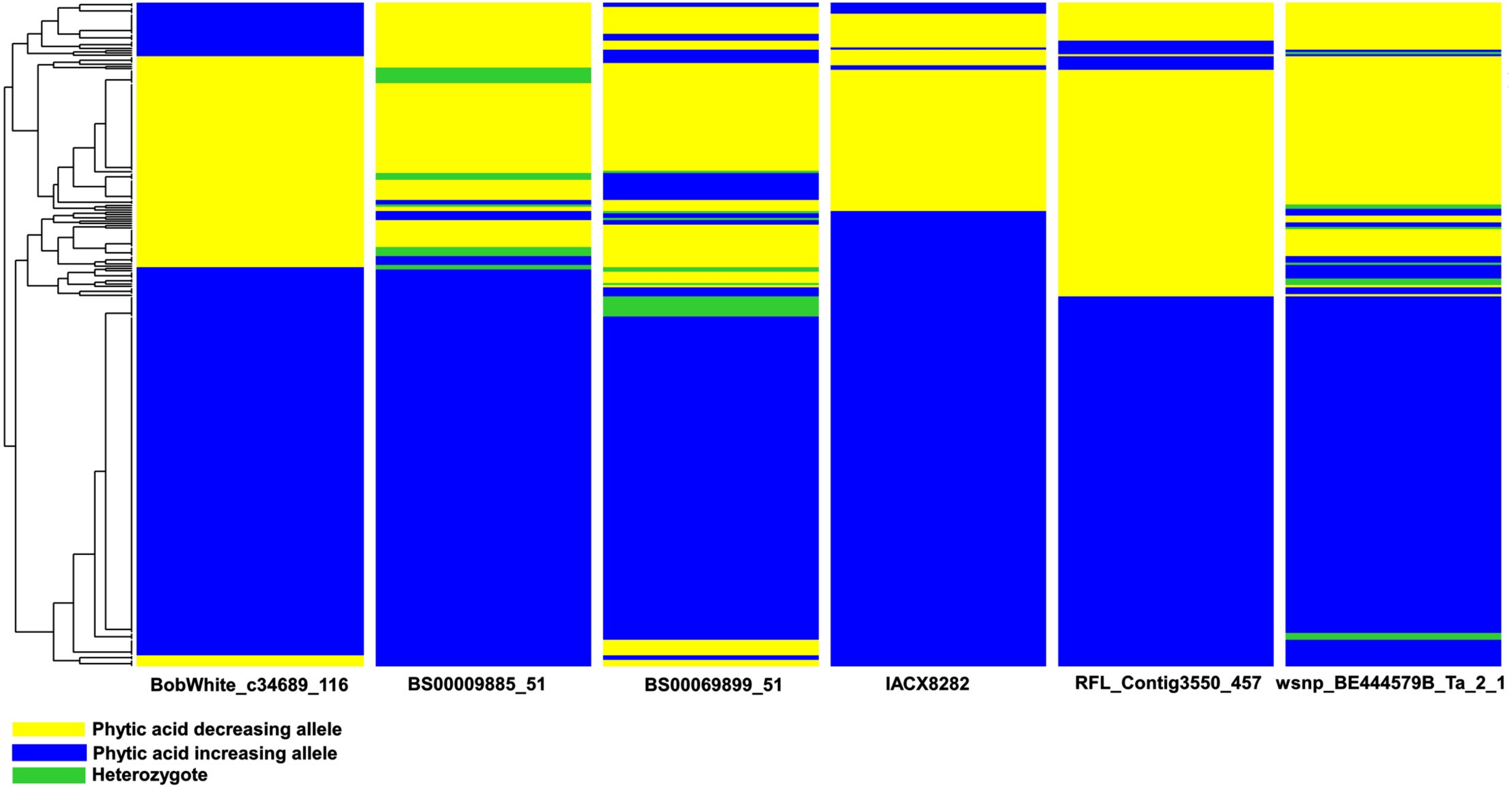

Supplementary Figure 10. Allelic fingerprinting and clustering of lines based on alleles at six consistent markers associated with grain phytic acid content.

The yellow color is for the favorable allele (allele that has a decreasing effect on the grain PA content), the blue color for the non-favorable allele (allele that has an increasing effect on the grain PA content), and the green color for the heterozygote.

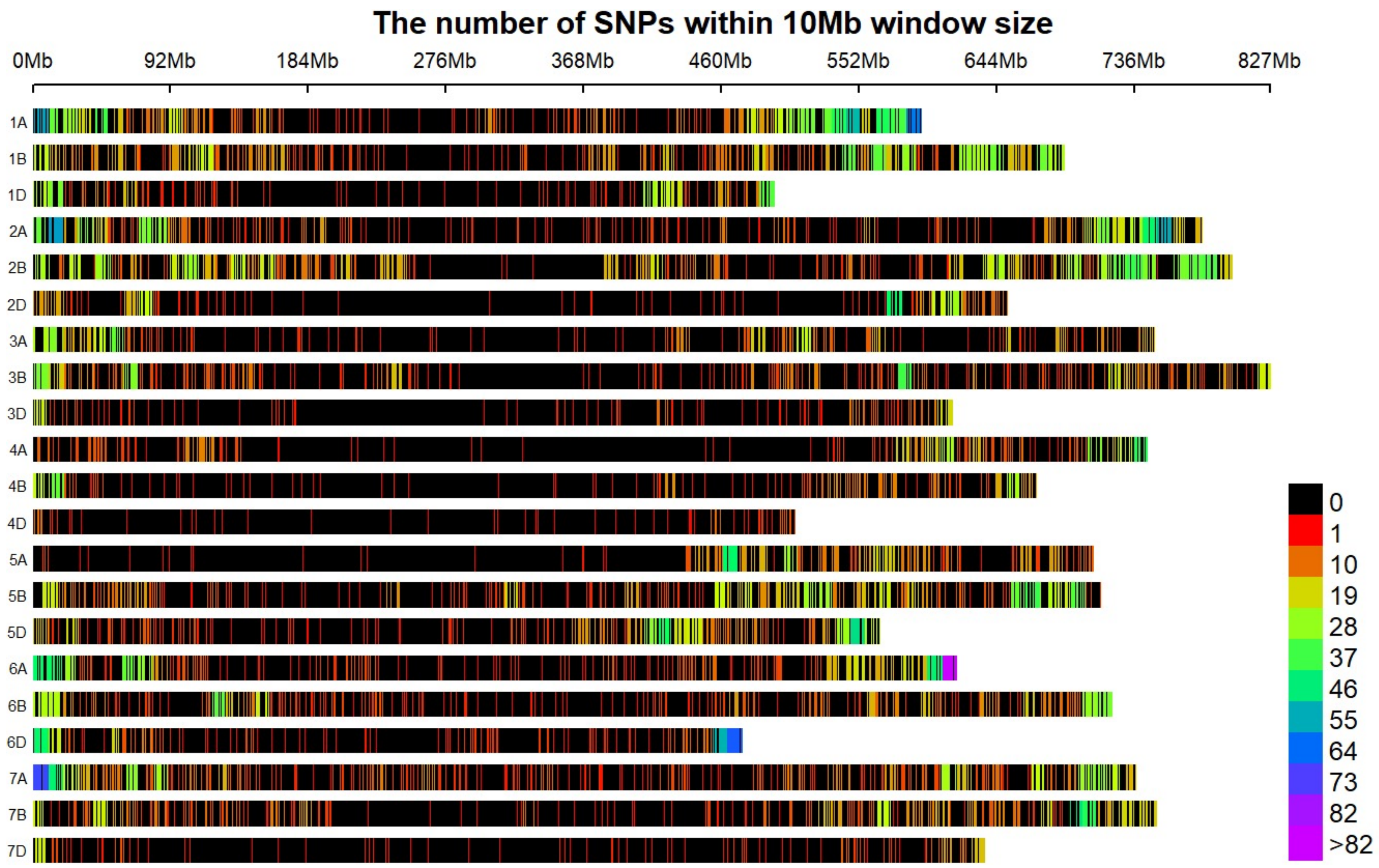

Supplementary Figure 11. Densities of the 10,158 single nucleotide polymorphisms (SNPs) used for GWAS showing the number of SNPs within 10 Mb window size.

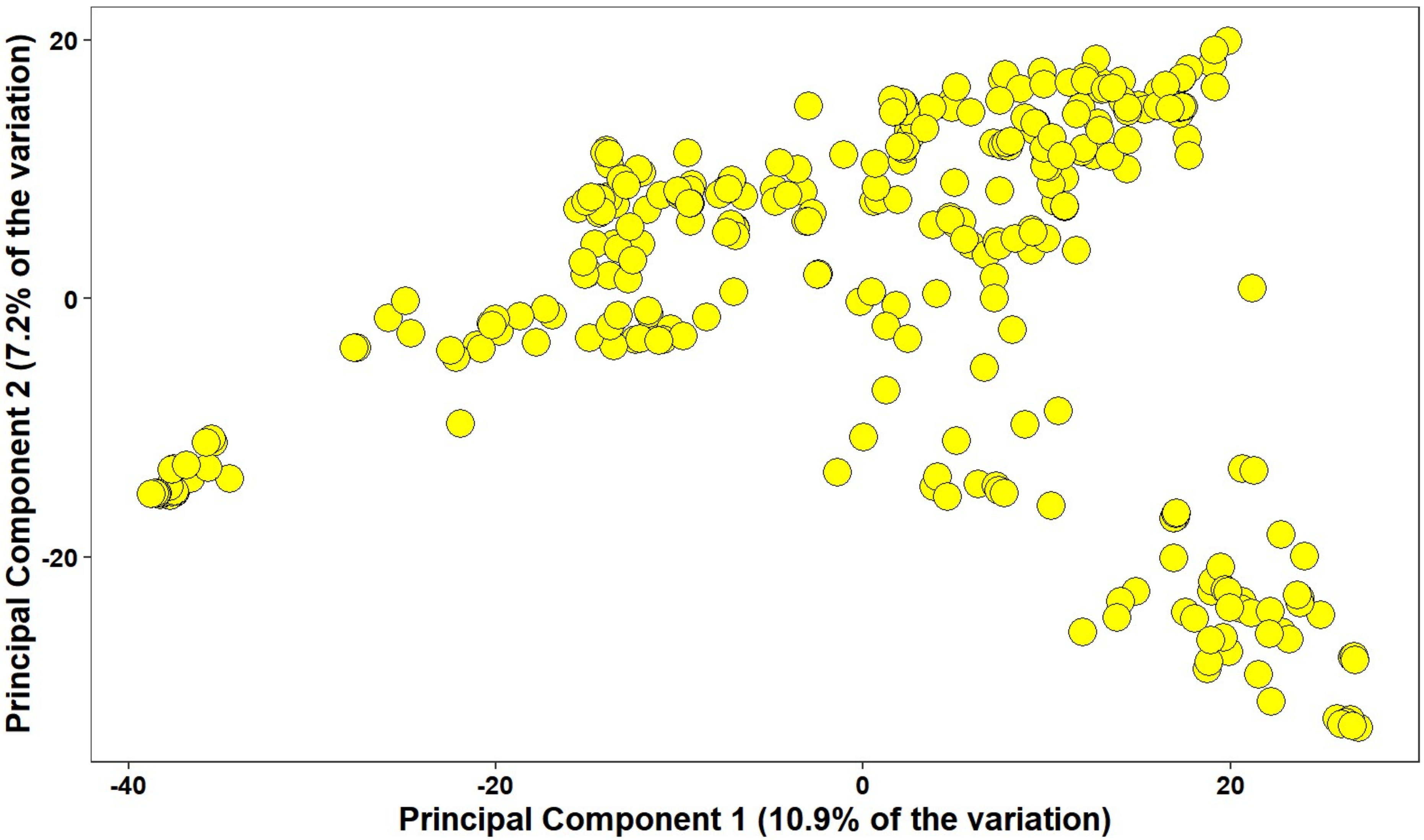

Supplementary Figure 12. Population structure analysis showing the plot of principal component 1 vs principal component 2 of the HPAM panel.

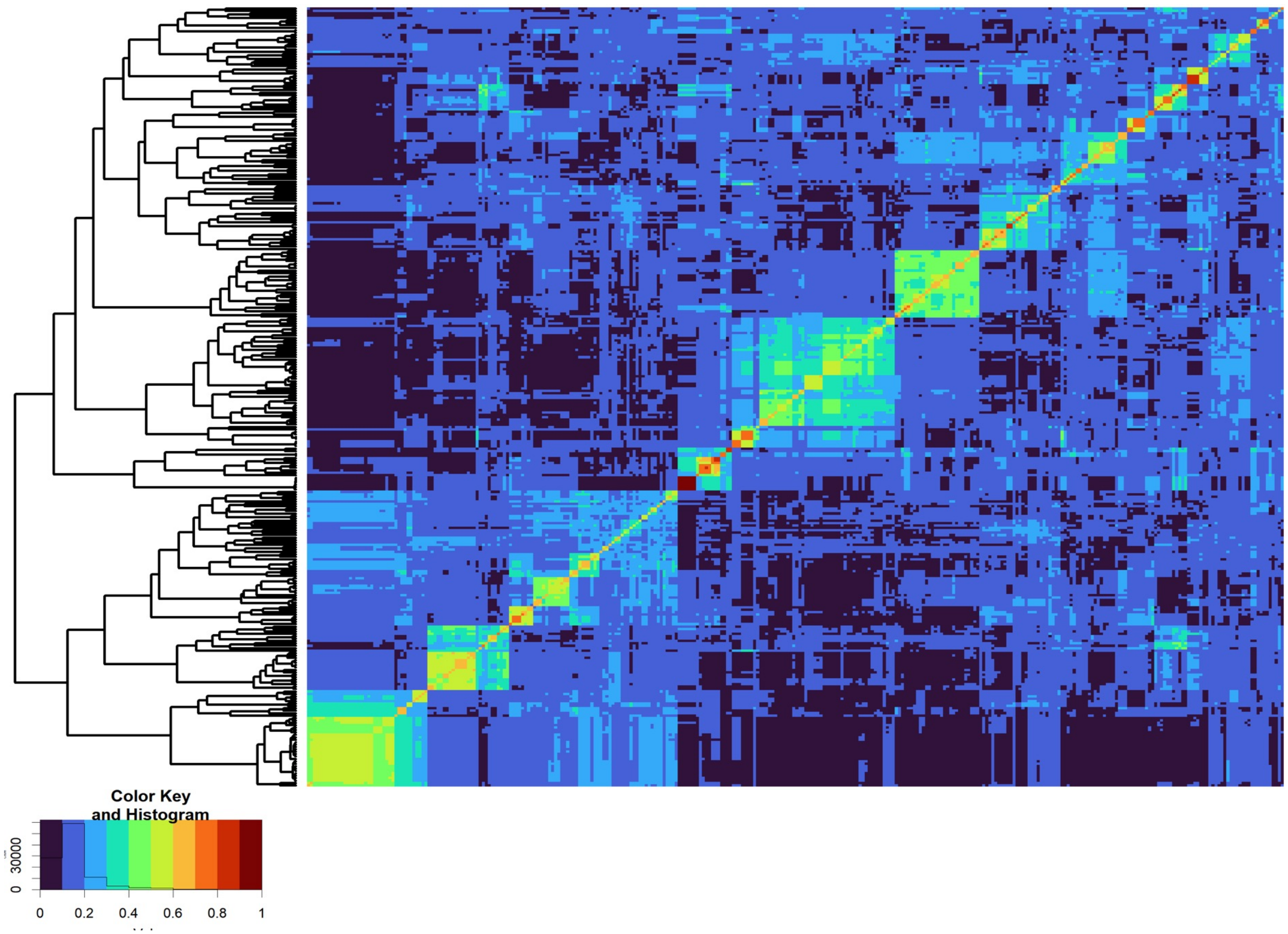

Supplementary Figure 13. Kinship analysis of the HPAM panel.
